# MTase Domain of *Dendrolimus punctatus cypovirus* VP3 Mediates Virion Attachment and Interacts with Host ALP Protein

**DOI:** 10.3390/v9040066

**Published:** 2017-04-01

**Authors:** Lan Su, Congrui Xu, Chuangang Cheng, Chengfeng Lei, Xiulian Sun

**Affiliations:** 1 Wuhan Institute of Virology, Chinese Academy of Sciences, Wuhan 430071, China; sul@wh.iov.cn (L.S.); xucr@wh.iov.cn (C.X.); ccg083@126.com (C.C.); cflei@wh.iov.cn (C.L.); 2 University of Chinese Academy of Sciences, Beijing 100049, China

**Keywords:** DpCPV, attachment proteins, ALP, viral entry

## Abstract

*Dendrolimus punctatus cypovirus* (DpCPV) is an important pathogen of *D. punctatus*, but little is known about the mechanisms of DpCPV infection. Here, we investigated the effects of VP3, VP4 and VP5 structural proteins on the viral invasion. Both the C-terminal of VP3 (methyltransferase (MTase) domain) and VP4 (A-spike) bound to *Spodoptera exigua* midgut brush border membrane vesicles (BBMVs) in a dose-dependent manner, and the binding was inhibited by purified DpCPV virions. Importantly, anti-MTase and anti-VP4 antibodies inhibited viral binding to *S. exigua* BBMVs. Using far-Western blots, a 65 kDa protein in *Bombyx mori* BBMVs, identified as alkaline phosphatase protein (*Bm*ALP) by mass spectrometry, specifically interacted with DpCPV MTase. The interaction between MTase and *Bm*ALP was verified by co-immunoprecipitation in vitro. Pretreatment of *B. mori* BBMVs with an anti-ALP antibody or incubation of DpCPV virions with prokaryotically expressed *Bm*ALP reduced viral attachment. Additionally, *Bm*ALP inhibited DpCPV infection in *S. exigua* larvae. Our data provide evidence that the MTase domain and A-spike function as viral attachment proteins during the DpCPV infection process, and ALP is the ligand that interacts with DpCPV via the MTase domain. These results augment our understanding of the mechanisms used by cypoviruses to enter their hosts.

## 1. Introduction

Cypovirus (CPV), a segmented double-stranded ribonucleic acid (dsRNA) virus within the genus *Cypovirus* (*Reoviridae* family), possesses a single capsid layer and is commonly embedded in the characteristic crystalline inclusion body called a polyhedron, which is formed in the cell cytoplasm of insects [1]. CPV infection occurs via the fecal-oral route and is mainly restricted to the larvae midgut. Accordingly, after the consumption of contaminated food by the larvae, the polyhedra dissolve in the highly alkaline environment of the midgut, releasing virions that adhere to and penetrate the plasma membrane of microvilli and settle in the cytoplasm of the columnar epithelial cells [2]. The recent study showed that CPV enter the cell by clathrin-mediated endocytosis employing integrin beta and receptor for activated protein kinase C (RACK1) [3]. Consequently, populations of progeny viruses are synthesized and embedded into a polyhedrin. As the disease progresses, the digestive and absorptive functions are severely affected and the larvae die one after another.

*Dendrolimus punctatus cypovirus* (DpCPV) is an important viral pathogen isolated from *D. punctatus*, with a relatively wide host range in *Lepidoptera*. In China, this virus species was developed in 2010 as a commercial insecticide to control the pine caterpillar, *D. punctatus*. Cryoelectron microscopy studies have documented the fine structures of the viral particles [4,5]. On the single capsid shell formed by VP1, three turret proteins exist: B-spikes formed by the C-terminal of VP3 (methyltransferase (MTase) domain), A-spikes formed by VP4 [6], and large protrusion proteins formed by VP5. The A-spike extends from the surface of the B-spike, but it is flexibly attached to the B-spike [5,7]. Examination of density slices indicates that the A-spike is directly bound to a polyhedron [5,6,7]. As the A-spike is the most projecting portion of CPV virions, and has a similar localization on the virion structure to the σ1 protein of mammalian orthoreovirus (MRV), it has been speculated that the CPV A-spike is probably associated with cell attachment and penetration [5,7,8]. Despite its precise functions in CPV infection, its interactions with host proteins have not been determined yet.

In general, viral attachment to one or more cellular receptors is the first step of viral invasion, and this process may determine the host range and cell tissue tropism of a virus [9,10]. It is believed that viral spike proteins perform essential functions in receptor binding and cell penetration, as seen with the VP7 protein of the bluetongue virus [9], the VP4 spikes of rotavirus [11] and the σ1 protein of MRV [12]. In *Reoviridae*, the entry details of reovirus and rotavirus have been well characterized. As the attachment protein of reovirus, an σ1 tail inserts into the virion via “turrets” formed by the λ2 protein, and its head projects away from the virion surface [13]. Reovirus initially tethers to sialylated glycans by the σ1 tail with low affinity, followed by higher affinity binding to junctional adhesion molecule A (JAM-A) by the σ1 head [14]. In the central nervous system, reovirus binds the Nogo receptor, NgR1, a leucine-rich repeat protein, to infect neurons [10]. The rotavirus cell entry is a multistep process. It is proposed that the rotavirus functional receptor is likely to be a complex of several cell components including gangliosides, integrins, and probably other proteins such as hsc70, which might be associated in lipid rafts and which need the lipid microdomain organization to efficiently mediate cell entry of rotaviruses [15].

In this study, we tested the binding characteristics of DpCPV turret proteins to midgut brush border membrane vesicles (BBMVs), followed by competition and neutralization assays. Furthermore, we showed that MTase and VP4 functioned as the viral attachment proteins in the DpCPV infection process and alkaline phosphatase protein (ALP) served as the MTase ligand.

## 2. Materials and Methods

### 2.1. Viruses, Larvae, and Antibodies

DpCPV was initially isolated from *D. punctatus* larvae in Macheng, Hubei, China, and propagated in *Spodoptera exigua* larvae. *S. exigua* and *Bombyx mori* larvae were obtained from the Core Facility and Technical Support, Wuhan Institute of Virology, Chinese Academy of Sciences. Polyclonal antibodies against the DpCPV virions, MTase domain of VP3 [16], VP4 [6] and VP5 [17] were prepared previously in our laboratory.

### 2.2. Recombinant Proteins

(i) Maltose binding protein (MBP)-fused DpCPV structural proteins. DpCPV VP3 was truncated to the galactosyltransferase (GTase) (1–366 aa) and MTase (406–1058 aa) domains based on its structure [5]. The gene segments encoding GTase, MTase, VP4, and VP5 were amplified from a complimentary deoxyribonucleic acid (cDNA) library [6] using the corresponding PCR primer pairs (Sangon, Shanghai, China) (listed in Table 1) and cloned into a pMal-C2X vector (NEB (Beijing), Beijing, China) as N-terminal MBP fusion proteins. The recombinant plasmids were transformed into *Escherichia coli* BL21 cells (Invitrogen, Carlsbad, CA, USA), and induced by isopropyl-β-d-thiogalactopyranoside for protein production. The soluble fusion proteins were purified by amylose resin column separation (NEB) according to the manufacture’s instruction manual.

(ii) *Bm*ALP. The truncated *alp* gene fragment without the transmembrane domain encoding amino acids 1–519 (GenBank: NM_001044071.3) was amplified from *B. mori* midgut total RNA by reverse transcriptase PCR with the primer pairs *alp*-F: 5′-CGGAATTCATGTCTACATGGTGGTTAGTTGTG-3′ and *alp*-R: 5′-CCCAAGCTTTTAGCGGCCCGGGC-3′ and then cloned into a pET28a vector (Invitrogen) with a 6 × His-tag at the C-terminal. The cell lysate from the transformed *E. coli* BL21 cells (Invitrogen) was purified by a Ni-nitrilotriacetic acid (NTA) agarose column (Invitrogen). Purified ALP was used to produce polyclonal antibodies in an ALP-immunized rabbit.

### 2.3. Purification of DpCPV Virions

DpCPV occlusion bodies (OBs) were isolated from infected *S. exigua* larvae by differential centrifugation. To obtain DpCPV virions, OB suspensions were lysed with 0.2 M Na_2_CO_3_-NaHCO_3_ (pH 10.8) and then pH was adjusted to 7.5 with 1 M Tris-Cl (pH 6.8), then purified by linear 20%–60% (*w/v*) sucrose gradient centrifugation [16]. The concentration of the purified virions was determined by a UV-31000PC spectrophotometer (Mapada, Shanghai, China) at 280 nm.

### 2.4. Preparation of BBMVs

Midgut brush border membrane vesicles (BBMVs) were prepared from the dissected midguts of fourth-instar *S. exigua* or *B. mori* larvae by the differential magnesium precipitation method [18] and then stored in MET buffer (0.3 M Mannitol, 17 mM Tris-HCl, 5 mM EGTA, pH 7.5) at −80 °C. The protein concentration was determined by the Bradford method with bovine serum albumin (BSA) as the standard.

### 2.5. Binding Characteristics of S. exigua BBMVs and Competition Assays

ELISA plates (96-well) were coated with 10 µg/mL of *S. exigua* BBMVs (*Se*BBMVs) in carbonate buffer at 4 °C overnight and blocked with 3% BSA for 2 h. Increasing amounts of purified DpCPV virions (0.01–100 µg) or MBP-fusion proteins (0.02–4 µM) in a total volume of 100 µL were added to the wells and incubated for 1 h. The wells were washed with PBST (4 mM KH_2_PO_4_, 16 mM Na_2_HPO_4_, 115 mM NaCl, 0.05% Tween 20, pH 7.4) and incubated with a rabbit anti-DpCPV virion antibody or a mouse anti-MBP mAb (Sigma-Aldrich, St. Louis, MO, USA) followed by horseradish peroxidase (HRP) labeled anti-rabbit IgG (Sigma-Aldrich) or HRP labeled anti-mouse IgG (Proteintech, Wuhan, China). The reactions were visualized using TMB reagent (Beyotime, Nantong, China), stopped by addition of 2 M H_2_SO_4_, and the absorbance was measured at 450 nm using an ELx808 absorbance reader (BioTek, Winooski, VT, USA). The non-specific binding of BSA coated wells, instead of BBMVs, was subtracted from the total binding value. For the competition assays, the concentrations of the MBP-fusion proteins were kept stable (0.3 µM), while the DpCPV virions were serially diluted (0–10 µg). The protein-virion mixtures were added to the wells; after absorption for 1 h, the bound MBP-fusion proteins were detected as described above.

### 2.6. Inhibition of Viral Binding by Antibodies

For the inhibition assays, DpCPV virions (final amounts, 10 μg each) were pretreated with different concentrations (1–100 μg/mL) of anti-MTase antibody, anti-VP4 antibody, a mixture of anti-MTase and anti-VP4 antibodies or the pre-immune antibody at 37 °C for 1 h. The individual mixtures were added to *Se*BBMVs (10 μg/mL) coated on 96-well ELISA plates. After absorption for 1 h, the bound virions were detected as described above. The binding rates for each concentration of the three antibody treatments were compared by One-way ANOVA followed by least significant difference (LSD) *t*-tests after Arcsin square root transformation.

### 2.7. Far-Western Blotting

*B. mori* BBMVs (*Bm*BBMVs) (10 μg/lane) were separated by 10% sodium dodecyl (lauryl) sulfate-polyacrylamide gel electrophoresis (SDS-PAGE), transferred to a polyvinylidene difluoride (PVDF) membrane (Millipore, Billerica, MA, USA) and blocked in 5% BSA in phosphate buffered saline (PBS) for 2 h. The membranes were incubated with 5 μg/mL of purified MBP-MTase, MBP-VP4, or MBP proteins (as the negative control) for 1 h at room temperature. After washing with PBST buffer, the membranes were incubated with a mouse anti-MBP mAb (Sigma-Aldrich) followed by HRP-labeled anti-mouse IgG (Proteintech), and visualized using ECL reagent (Beyotime).

### 2.8. Mass Spectrometry Analysis

A coomassie brilliant blue stained protein band of 65 kDa was excised from a 10% SDS-PAGE gel and subjected to in-gel digestion using trypsin at 37 °C overnight. Liquid chromatography- tandem mass spectrometry (LC-MS/MS) was performed, and the obtained peptide sequences were analyzed by the MASCOT program based on the database of *Bombyx*.

### 2.9. Co-Immunoprecipitation (Co-IP) Assays

Protein G Dynabeads (Thermo Scientific, Waltham, MA, USA) (20 μL) were immobilized with 5 μL of mouse anti-MBP mAb (Sigma-Aldrich) overnight at 4 °C, mixed with 20 μg of MBP or MBP-MTase with 20 μg of ALP or BSA in a total volume of 500 μL PBS and then incubated at 4 °C for 1 h. The beads were washed five times with 500 μL of PBS with a magnet, suspended in 30 μL of 2 × sodium dodecyl (lauryl) sulfate (SDS) sample buffer, boiled for 5 min, and loaded on a 10% SDS-PAGE gel for Western blotting analysis using a mixture of mouse anti-MBP (Sigma-Aldrich) and mouse anti-His (Proteintech) mAbs. Prokaryotically expressed *Bm*ALP protein was used as the control.

### 2.10. Inhibition of Viral Binding

(i) By anti-ALP antibody. *Bm*BBMVs proteins (10 µg/mL) coated on 96-well ELISA plates were pretreated with different concentrations of the anti-ALP or pre-immune antibodies (0.2–20 µg/mL) at 37°C for 1 h, then incubated with DpCPV virions (final amounts, 10 µg) at 37 °C for a further 1 h. The wells were washed with PBST and the bound virions were detected using a rabbit anti-DpCPV virion antibody followed by HRP-labeled anti-rabbit IgG (Sigma-Aldrich).

(ii) By *Bm*ALP proteins. DpCPV virions (final amounts, 10 µg) were pretreated with different concentrations of *Bm*ALP protein (0.02–2 µM) or BSA at 37 °C for 1 h, and then added to the *Bm*BBMVs (10 µg/mL) coated in 96-well ELISA plates. After absorption for 1 h, the bound virions were detected as described above.

### 2.11. In Vivo Neutralization Tests

Neutralization bioassays were conducted using a modified version of the droplet-feeding method [19]. Second instar *S. exigua* larvae were starved for 16 h before treatment. The larvae were divided into four groups: group 1 were fed PBS, group 2 DpCPV virions, group 3 DpCPV virions plus BSA, and group 4 DpCPV virions plus *Bm*ALP. The final amount of DpCPV virions was 10 µg in all the groups, and the concentration of *Bm*ALP or BSA proteins was 20 µg/mL. The virion-protein mixtures were incubated for 1 h, and then mixed with 40% sucrose and Erioglaucine disodium salt (food coloring) in a total volume of 1 mL. The larvae fed with the resulting mixture were judged by the uptake of the blue stain, and then transferred to the individual wells of a 24-well culture plate containing fresh artificial diet. The experiment was conducted with 48 larvae in each group and replicated in duplicate. The number of dead insects was recorded every day until all the larvae died or pupated (15 days post inoculation). The survival function of the larvae among the groups was estimated by the Kaplan-Meier method and compared using the log-rank test [20]. The data from two replicates were pooled for survival analysis as long as the two replicates did not differ significantly.

## 3. Results

### 3.1. DpCPV Virions Binding to SeBBMVs

**Figure 1:**
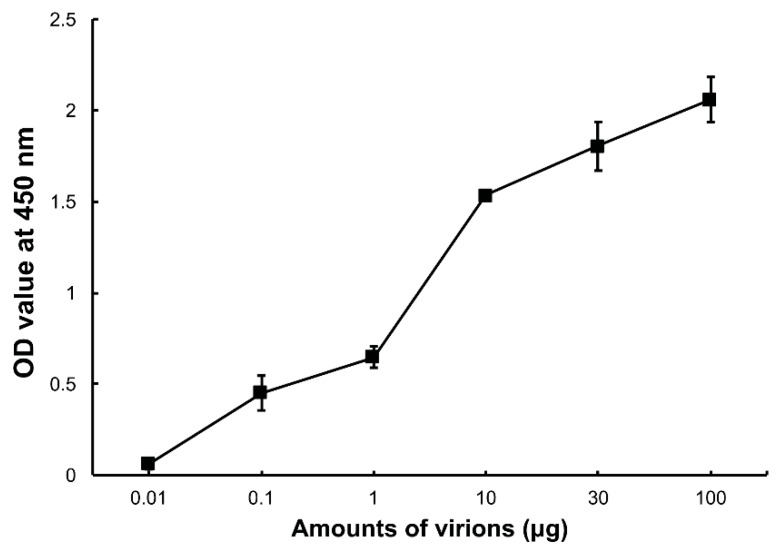
Binding of *Dendrolimus punctatus cypovirus (*DpCPV) virions to *Spodoptera exigua* midgut brush border membrane vesicles (*Se*BBMVs). *Se*BBMVs (10 µg/mL) were coated on 96-well plates and incubated with the amount of purified DpCPV virions indicated. Virion binding was determined using a rabbit anti-DpCPV virion antibody followed by horseradish peroxidase (HRP)-labeled anti-mouse IgG as the secondary antibody. Nonspecific binding was determined with bovine serum albumin (BSA) coated wells instead of those coated with BBMVs.

To confirm whether DpCPV virions can bind to *Se*BBMVs, an ELISA was performed in which increasing amounts of the purified virions were incubated with *Se*BBMVs coated on 96-well ELISA plates. The results showed that the DpCPV virions bound to the *Se*BBMVs in a dose-dependent manner (Figure 1), and no binding was observed when BSA was the coating control.

### 3.2. Binding Characteristics of the MBP Fusion Proteins to SeBBMVs

**Figure 2:**
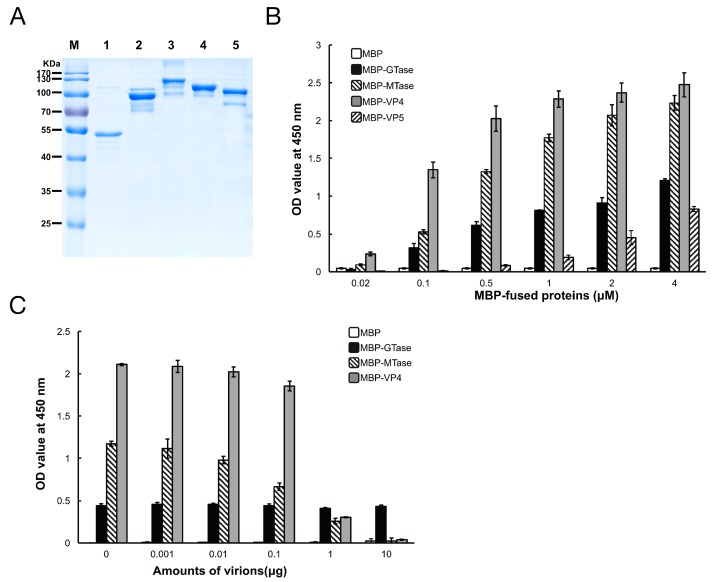
Maltose binding protein (MBP)-fusion protein binding to *Se*BBMVs. (**A**) Expression and purification of MBP-fusion proteins. MBP (lane 1), MBP-galactosyltransferase (GTase) (lane 2), MBP-methyltransferase (MTase) (lane 3), MBP-VP4 (lane 4), and MBP-VP5 (lane 5) were separated by 12% sodium dodecyl (lauryl) sulfate-polyacrylamide gel electrophoresis (SDS-PAGE); (**B**) *Se*BBMV proteins were coated on 96-well plates and incubated with the concentrations of MBP-fusion proteins indicated. The binding efficiency was determined with an anti-MBP mAb and HRP-labeled IgG as the secondary antibody. Nonspecific binding was determined with BSA coating instead of *Se*BBMVs; (**C**) MBP-fusion proteins (0.3 µM) were mixed with the amounts of purified DpCPV virions indicated, then added to the *Se*BBMVs-coated 96-well plates, and absorption was allowed for 1 h. Protein binding was determined as described above.

To determine which protein in DpCPV mediates efficient binding to susceptible host midgut BBMVs, the GTase and MTase domains of VP3, and the full-length VP4 and VP5 proteins were co-expressed with MBP as soluble proteins along with MBP. The sizes of purified MBP, MBP-GTase, MBP-MTase, MBP-VP4, and MBP-VP5 were 42 kDa, 94 kDa, 123 kDa, 112 kDa, and 98 kDa, respectively (Figure 2A), which is consistent with the predicted sizes of these proteins plus the N-terminal MBP.

Next, the binding efficiency of the DpCPV turret proteins to *Se*BBMVs was tested. MBP-GTase, MBP-MTase, and MBP-VP4 binding to *Se*BBMVs were all dose-dependent, while MBP-VP5 bound at a relatively low level (Figure 2B). No binding was detected with the MBP-incubated wells. To further test the binding specificity, GTase, MTase, or VP4 proteins were mixed with different amounts of purified DpCPV virions and then the mixtures were added to the *Se*BBMVs. This treatment reduced the binding of MBP-MTase and MBP-VP4 in a dose-dependent manner (Figure 2C), suggesting that the MTase domain and VP4 competed with the virions for the same cellular receptor. In contrast, MBP-GTase binding to the *Se*BBMVs was unaffected by virion addition (Figure 2C).

### 3.3. Inhibition of Viral Binding by Anti-MTase and Anti-VP4 Antibodies

**Figure 3:**
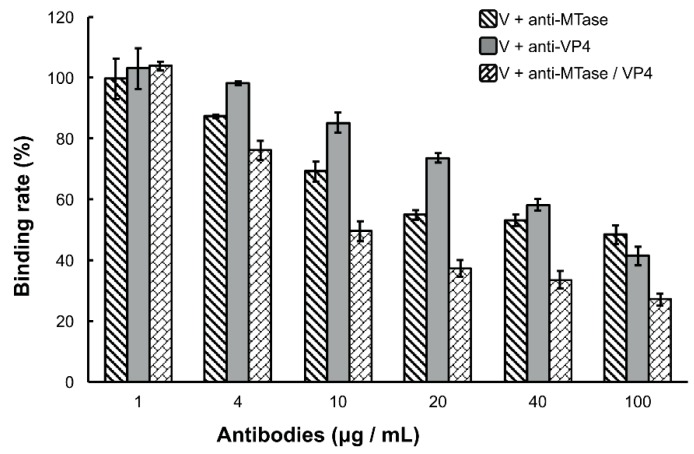
Inhibition of viral binding by anti-MTase and/or anti-VP4 antibodies. Purified DpCPV virions (fixed amounts of 10 µg) were pretreated with the concentrations of different antibodies indicated before inoculation into *Se*BBMVs (10 µg/mL) and coated on 96-well ELISA plates. Viral binding was determined by ELISA as described above. No significant differences were observed between the virion alone treatment and the virion with pre-immune antibody treatment regardless of the dilution factor. The error bars represent standard deviations.

Because the DpCPV virions bound specifically to the *Se*BBMVs, we next tested whether the viral attachment could be blocked by antibodies to MTase and/or VP4. For this, purified virions (fixed 10 µg amounts) were incubated with different concentrations of anti-MTase and/or anti-VP4 antibodies for 1 h prior to inoculation to the *Se*BBMVs. At all higher concentration treatments (4, 10, 20, 40, and 100 µg/mL), the mixture of anti-MTase and anti-VP4 antibodies reduced the viral attachment more effectively than the antibodies used alone (F_2, 6_ = 60.3, 93.7, 245.1, 78.3, and 38.7, respectively, *p* < 0.001; Figure 3). For example, at the highest concentration of antibodies (100 µg/mL), while the single anti-MTase or anti-VP4 antibodies inhibited viral attachment to 48.4% ± 3.1% and 41.4% ± 3.9%, respectively, the mixed antibodies inhibited viral attachment to 27.2% ± 1.9% in comparison with the control (pre-immune antibody); this treatment was significantly more effective than the anti-MTase antibody (LSD-*t* = 8.607, *p* = 0.00014) or the anti-VP4 antibody alone (LSD-*t* = 5.676, *p* = 0.00108).

### 3.4. Identification of MTase Binding Proteins in Host BBMVs

**Figure 4:**
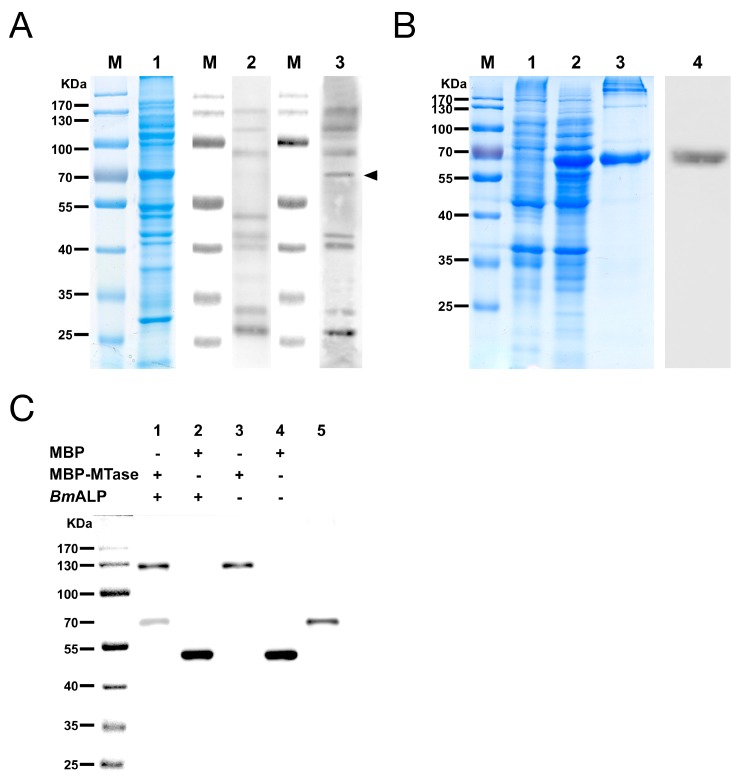
The MTase binding protein was identified as *B. mori* alkaline phosphatase protein (ALP). (**A**) Far-Western blotting of *Bm*BBMVs with MBP-MTase. Blot of *Bm*BBMVs were separated by 10% SDS-PAGE (lane 1), incubated with MBP (lane 2) or MBP-MTase (lane 3), and probed with an anti-MBP mAb followed by HRP-labeled anti-mouse IgG as the secondary antibody; (**B**) Expression and purification of recombinant *Bm*ALP protein separated by 12% SDS-PAGE. Lanes: M, protein molecular mass marker; 1, non-induced bacterial cell lysate; 2, induced bacterial cell lysate; 3, *Bm*ALP purified by a Ni-NTA agarose column; 4, *Bm*ALP detected with a rabbit anti-ALP antibody; (**C**) Co-immunoprecipitation (Co-IP) validated the interaction between MBP-MTase and *Bm*ALP. The immunoprecipitated products (lane 1 to lane 4) and *Bm*ALP protein (lane 5) were separated by SDS-PAGE, transferred to a PVDF membrane, and were incubated with a mixture of mouse anti-MBP mAb and mouse anti-His mAb as the primary antibody and HRP-labeled anti-mouse IgG as the secondary antibody.

Far-Western blotting was utilized to identify the host proteins that bound to DpCPV MTase and VP4. A 65 kDa protein from *Bm*BBMVs, which bound to MBP-MTase, was detected, while other membrane proteins also bound to the MBP protein control in a parallel assay (Figure 4A). However, MBP-VP4 showed no interaction with *Bm*BBMVs under the same conditions. The 65 kDa protein was excised from the gel and analyzed by mass spectrometry, the identified result is shown in Table 2. Since *B. mori* ALP (gi|189332880) was consistent with the protein molecular weight of 65 kDa and was a component of midgut membranes, *Bm*ALP was further investigated. Recombinant *Bm*ALP (1–519 aa; without the transmembrane domain) was expressed in *E. coli* (Figure 4B), a rabbit anti-ALP antibody was raised and Western blotting showed that the anti-ALP antibody reacted with *Bm*ALP.

The interaction between MBP-MTase and *Bm*ALP was validated by Co-IP. The results showed that *Bm*ALP was only co-precipitated following incubation with MBP-MTase, not MBP alone (Figure 4C, lanes 1 and 2), suggesting that MTase bound directly to *Bm*ALP.

### 3.5. Inhibition of Viral Binding to BmBBMVs

**Figure 5:**
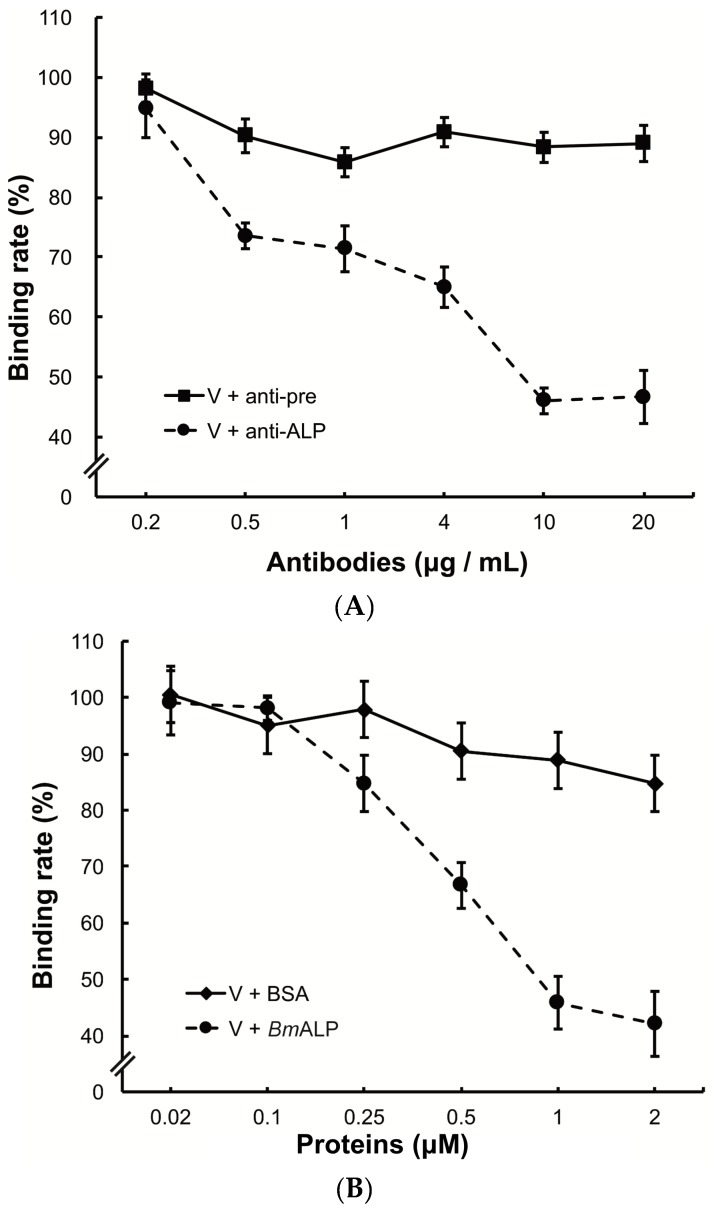
Inhibition of viral binding by anti-ALP antibody or *Bm*ALP protein. (**A**) *Bm*BBMVs (10 µg/mL) coated on 96-well plate were incubated with different concentrations of anti-ALP or pre-immune antibodies (0.2–20 µg/mL). After washing, DpCPV virions (10 µg) were added to each well. Viral binding was determined as described in the main text; (**B**) DpCPV virions (10 µg) were pre-incubated with different concentrations of *Bm*ALP or BSA protein (0.02–2 µM) before inoculation into the *Bm*BBMVs-coated 96-well plate. Viral binding was determined as described in the main text.

To investigate the function of ALP in DpCPV binding, *Bm*BBMVs coated in the ELISA plate were pretreated with different concentrations of the anti-ALP antibody prior to viral inoculation. Treatment with the anti-ALP antibody significantly reduced the attachment of DpCPV virions in a dose-dependent manner, and the final rate was 46.7% ± 3.0% of the control where no antibody was added (Figure 5A). In contrast, pre-immune antibody treatment did not reduce viral attachment.

To further study the role of ALP, DpCPV virions were incubated with different concentrations of *Bm*ALP before inoculation into *Bm*BBMVs. The results showed that viral binding was reduced as the concentration of *Bm*ALP increased, and the final rate was 42.1% ± 5.8% of the control where no protein was added (Figure 5B). However, BSA (as a control) had no effect on the binding.

### 3.6. In Vivo Neutralization with ALP in S. exigua

**Figure 6:**
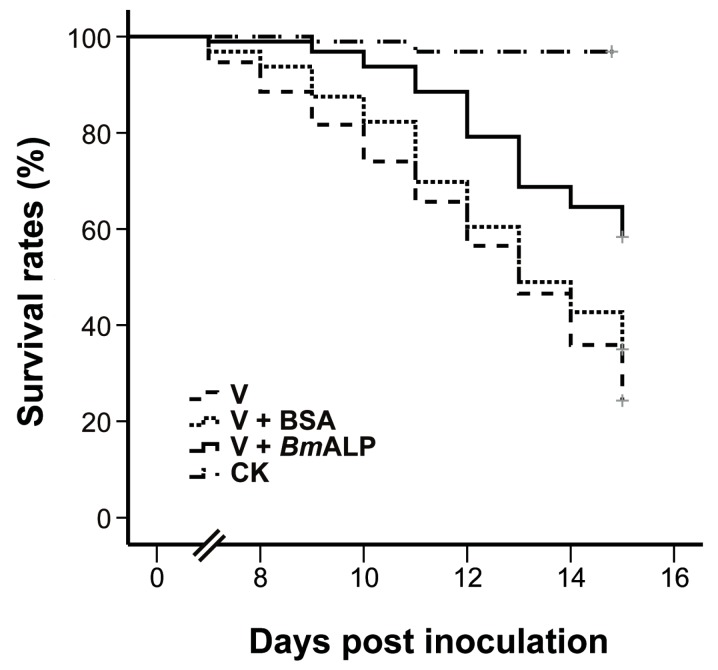
In vivo neutralization with *Bm*ALP in *S. exigua*. Survival rate data represent the results pooled from two replications (*n* = 96 for each treatment).

To ascertain whether ALP mediates DpCPV infectivity, an in vivo neutralization test was performed. Purified DpCPV virions were incubated with increasing concentrations of *Bm*ALP prior to inoculation of *S. exigua*. At 4 days post inoculation, the larvae started to exhibit signs of CPV infection, such as appetite loss, slow movement, and bradygenesis. From days 7 to 15 post inoculation, the survival rates of treated larvae decreased steadily, and the final survival rates of larvae treated with virions alone and virions plus BSA were 33.3% and 34.4%, respectively (*n* = 96) (Figure 6). However, the survival rate of larvae treated with virions plus *Bm*ALP was 58.3% (*n* = 96). Overall, the survival function of the larvae treated with virions and *Bm*ALP was significantly different from that of the larvae treated with virions alone (χ^2^ = 27.274, *p* < 0.0001) or virions plus BSA (χ^2^ = 12.822, *p* = 0.000343). The dead larvae did not liquefy and their cuticles were intact, displaying a typical feature of CPV infection. In contrast, most larvae pupated in the PBS group. Although *Bm*ALP could not completely block DpCPV infection in *S. exigua*, the results imply that ALP contributes to the infectivity of DpCPV in larvae.

## 4. Discussion

CPVs, the second major group of insect viral pathogens, have been isolated from more than 250 different insect species reared in laboratories or from the field. In recent years, studies of CPV have focused extensively on its characterization and structure [5,21,22], but little is known about the molecular mechanisms of CPV infection because a reliable cell culture system to measure viral infection efficiency is lacking. In this study, we used midgut BBMV proteins instead of susceptible cell lines. Based on the consensus that spike proteins are the typical sites for viral interaction with host cells [9,11,12], we selected the turret proteins of DpCPV for investigation.

Initially, we confirmed that DpCPV particles could bind to *Se*BBMVs (Figure 1). Then, we found that the MTase domain of VP3 (B-spike) and VP4 (A-spike) bound efficiently to the *Se*BBMVs, whereas VP5 binding was at a relatively low level (Figure 2B). The structural homolog proteins of CPV VP5 are commonly present in the inner capsids of other turreted reoviruses (e.g., orthoreovirus λ2 and aquareovirus VP6), and they also function as molecular clamps to stabilize the inner capsids [23]. Furthermore, the competition assays (Figure 2C) suggested that the C-terminal of VP3 (MTase) rather than the N-terminal (GTase) participates in viral attachment to host midguts. This finding is consistent with the fact that the GTase domain is an interior turret part situated underneath the capsid shell [1,4], making it inaccessible to host molecules during the virus entry procedure. The potential roles for MTase and VP4 proteins in DpCPV attachment were investigated by blocking tests (Figure 3). The results showed that the mixture of anti-MTase and anti-VP4 antibodies was more effective at blocking the viral attachment than the anti-MTase and anti-VP4 antibodies used alone, indicating that both MTase (B-spike) and VP4 (A-spike) were involved in the viral attachment.

In vivo, DpCPV can infect a wide range of insects of different genera and the infections are mostly limited to the midgut [24]. These characteristics suggest that DpCPV can bind to common targets on midgut cells in a variety of hosts. Thus, we conducted far-Western blotting to identify the host factors that interact with DpCPV MTase and VP4. *Bm*BBMVs was used because *B. mori* was a model organism with a clear genetic background and could be infected by DpCPV. A 65 kDa protein in *Bm*BBMVs showed a specific interaction with MTase (Figure 4A) and was later identified as a glycosylphosphatidylinositol (GPI)-anchored ALP of *B. mori*. However, we failed to identify the MTase binding protein in *Se*BBMVs using the same conditions. Because ALP is a housekeeping gene in insects [25] and *B. mori* ALP shares 65% amino-acid sequence identity with *S. exigua* ALP (KM048197.1), we investigated *B. mori* ALP further.

ALP is a glycoprotein anchored to the cell membrane by a GPI anchor. It is abundant in midgut BBMVs and is estimated to account for 15 to 20% of the total midgut protein [25]. Immunofluorescence studies [26] have shown that ALPs are located primarily in the posterior midgut epithelial cells, the region in which DpCPV infection occurs. ALP is found at higher levels in the first and second larval instars [27], and it appears that in early instars, ALP plays an important role in the viral infection. It has been reported that it acts as a receptor for *Bacillus thuringiensis* Cry1Ac in *Manduca sexta* and *Heliothis virescens*, and as a Cry11Aa receptor in *Aedes aegypti* [25].

In the current study, Co-IP assays verified that *Bm*ALP specifically bound to the MTase (Figure 4C). Importantly, pretreatment of *Bm*BBMVs with an anti-ALP antibody or incubation of DpCPV virions with soluble *Bm*ALP resulted in significantly reduced attachment of virions to *Bm*BBMVs (Figure 5), indicating that ALP affected the attachment of DpCPV to *Bm*BBMVs. The results of in vivo neutralization assays (Figure 6) suggested that ALP was essential in the early steps of DpCPV infection. However, ALP on its own is not able to completely block viral attachment; thus, we presume that additional host factors act in concert with ALP to promote viral infection. For EV71, human P-selectin glycoprotein ligand-1 and human scavenger receptor class B member 2 (hSCARB2) have been identified as receptors. SCARB2 is expressed ubiquitously on most susceptible cell types. However, antibody blocking of SCARB2 can only partially reduce the entry of EV71 into some cell lines [28,29]. A more recent study found that Annexin II, a cellular adherent factor, also interacts with EV71 and enhances viral infectivity [30].

Based on our data, we hypothesize that DpCPV may enter host cells under the combined effects of A-spikes and B-spikes. Initially, DpCPV may attach to an unknown cellular receptor through A-spikes and then, following polyhedra disruption in the midgut, the A-spikes fall away from the B-spike of the virus particle. When this occurs, a more stable B-spike is exposed, and this attaches to the secondary receptor (ALP) on the midgut membrane. This entry pattern is similar to reoviruses, which rely on the coordinated engagement of multiple receptors to mediate the initial viral entry [10,13].

In summary, this is the first study to show that both the MTase domain (B-spike) and VP4 (A-spike) are responsible for CPV attachment. We also found that MTase interacted with *Bm*ALP, and this interaction affected the viral infection, although other host factors may also contribute to infection with DpCPV. In this regard, it would be pertinent to investigate the functional receptor for VP4 and how ALP, in conjunction with the additional receptor, behaves during CPV entry into cells. Our data facilitate better understanding of CPV pathogenesis and open the way for novel methods to study the interactions between CPV and hosts.

## Figures and Tables

**Table 1:** Primers used in this study.

Primer	Primer Sequence (5′→3′)
gtase-F	CGGGATCCATGTGGCATTATACGAGTATCAAC (*BamH*I Site underlined)
gtase-R	CCCAAGCTTTTAGGAAATATAATTCGCGGTGAT (*Hind*III Site underlined)
mtase-F	CGGAATTCGAGCCAATGAGCATAGCT (*EcoR*I Site underlined)
mtase-R	CGGGATCCTTACGAGCGTACAAGTTTGATC (*BamH*I Site underlined)
vp4-F	CGGAATTCATGTTCGCAATCGATCCACT (*EcoR*I Site underlined)
vp4-R	CGGAATTCATGTTCGCAATCGATCCACT (*BamH*I Site underlined)
vp5-F	CGGAATTCATGTTACAACAACCAGCAGGA (*EcoR*I Site underlined)
vp5-R	CGGGATCCTCATAGGATGTCATCTGAGTGC (*BamH*I Site underlined)

**Table 2:** Identification of host proteins by LC-MS/MS and MASCOT program.

Accession	Similar Protein (Protein Name/Organism)	MASCOT Score	Nominal Mass (Da)	Sequence Coverage (%)
gi|3721840	aminopeptidase N/Bombyx mori	41	109,624	16
gi|227072221	troponin I variant D/Bombyx mandarina	38	23,711	7
gi|356582739	TSSK/Bombyx mori	33	39,640	9
gi|189332880	alkaline phosphatase/Bombyx mori	32	60,682	9
